# Epidemiology of Pelvic Fractures in Germany: Considerably High Incidence Rates among Older People

**DOI:** 10.1371/journal.pone.0139078

**Published:** 2015-09-29

**Authors:** Silke Andrich, Burkhard Haastert, Elke Neuhaus, Kathrin Neidert, Werner Arend, Christian Ohmann, Jürgen Grebe, Andreas Vogt, Pascal Jungbluth, Grit Rösler, Joachim Windolf, Andrea Icks

**Affiliations:** 1 Department of Public Health, Faculty of Medicine, Heinrich-Heine University, Düsseldorf, Germany; 2 mediStatistica, Neuenrade, Germany; 3 AOK NordWest, Dortmund, Germany; 4 Coordination Centre for Clinical Trials, Faculty of Medicine, Heinrich-Heine University, Düsseldorf, Germany; 5 Department of Trauma and Hand Surgery, University Hospital, Düsseldorf, Germany; 6 Joint Practice for Diagnostic Radiology and Nuclear Medicine, Köln-Kalk, Germany; Garvan Institute of Medical Research, AUSTRALIA

## Abstract

Epidemiological data about pelvic fractures are limited. Until today, most studies only analyzed inpatient data. The purpose of this study was to estimate incidence rates of pelvic fractures in the German population aged 60 years or older, based on outpatient and inpatient data. We conducted a retrospective population-based observational study based on routine data from a large health insurance company in Germany. Age and sex-specific incidence rates of first fractures between 2008 and 2011 were calculated. We also standardized incidence rates with respect to age and sex in the German population. Multiple Poisson regression models were used to evaluate the association between the risk of first pelvic fracture as outcome and sex, age, calendar year and region as independent variables. The total number of patients with a first pelvic fracture corresponded to 8,041 and during the study period 5,978 insured persons needed inpatient treatment. Overall, the standardized incidence rate of all first pelvic fractures was 22.4 [95% CI 22.0–22.9] per 10,000 person-years, and the standardized incidence rate of inpatient treated fractures 16.5 [16.1–16.9]. Our adjusted regression analysis confirmed a significant sex (RR 2.38 [2.23–2.55], p < 0.001, men as reference) and age effect (higher risk with increasing age, p < 0.001) on first fracture risk. We found a slight association between calendar year (higher risk in later years compared to 2008, p = 0.0162) and first fracture risk and a further significant association with region (RR 0.92 [0.87–0.98], p = 0.006, Westfalen-Lippe as reference). The observed incidences are considerably higher than incidences described in the international literature, even if only inpatient treated pelvic fractures are regarded. Besides which, non-inclusion of outpatient data means that a relevant proportion of pelvic fractures are not taken into account. Prevention of low energy trauma among older people remains an important issue.

## Introduction

Pelvic fractures are one of the main results of low energy trauma such as falls, particularly in older individuals [1–4]. Due to the increase of the older population worldwide [5] the burden of pelvic fractures will become highly relevant for society in general and in particular for our healthcare systems. Consequently, low energy fractures are assumed to affect a growing number of individuals and an increase of pelvic fracture incidences has already been reported [3, 6–10]. Pelvic fractures are associated with significant morbidity and mortality [2, 11–13]; for instance, one year mortality after pelvic fractures is reported to be fairly substantial, ranging from about 8%-27% [2, 11, 12, 14, 15]. In addition, pelvic fractures will result in rising healthcare costs due to the requirement of hospital and follow-up care [7, 16–18]. In comparison to hip fractures, which have been thoroughly investigated, pelvic fractures have as yet only been analyzed to some extent. Even more striking, incidences of pelvic fractures show opposing trends in the older population compared with rates of hip fractures: while absolute numbers of hip fractures increase due to the aging of the population, age-standardized incidence rates are levelling off or even declining in a number of countries [19–23]. In contrast, age-standardized rates of pelvic fractures have also been found to increase in the last decades. There is sufficient evidence that incidences of pelvic fractures increase with age and are more common in women than in men. However, most of the available studies have focused on inpatient data, e.g. hospital admission or discharge diagnoses, but it can be assumed that a significant proportion of individuals with pelvic fracture are treated as outpatients [12, 24, 25]. Furthermore, most studies had no access to individual patient data and hence could not avoid double counting which may occur not only due to further fractures, but also due to hospital changes or readmissions because of complications. The aim of this study was to estimate incidence rates of pelvic fractures in the German population aged 60 years or older based on outpatient and inpatient data from a statutory health insurance. We further evaluated the association between the risk of first pelvic fracture as the dependent variable and sex, age, calendar year and region as independent variables.

## Methods

### Ethics Statement

The study was approved by the ethics committee of the Faculty of Medicine, Heinrich-Heine University Düsseldorf. The survey and utilization of secondary health administration data was conducted retrospectively and in compliance with the applicable standards and legal rules on data protection. All procedures performed were in accordance with the Declaration of Helsinki and comparable ethical standards (e.g., Good Epidemiologic Practice (GEP) [26] and Good Practice of Secondary Data Analysis (GPS) [27]). The data were analyzed anonymously; informed patient consent is not required.

### Study Design, data source and population

The study is a retrospective population-based observational study. Routine data for outpatient and inpatient care was provided by a large statutory health insurance company in Germany, the AOK NordWest. Overall, the AOK NordWest covers about 2.8 million insured people in two regions Schleswig-Holstein (700,000 insured) and Westfalen-Lippe (2.1 Million insured), of whom about 29% count 60 years or older. We included all people aged minimum 60 years who were continuously insured for at least one year between January 1, 2007 and December 31, 2011. The selection process is presented in detail below. Most persons were insured during the whole study period with the AOK NordWest.

### Ascertainment of pelvic fracture events

Pelvic fractures along with the exact date of occurrence were identified in inpatient and outpatient data according to the 10th revision of the International Classification of Diseases (ICD-10). A fracture event is defined by the ICD 10 codes S32.1 (fracture of sacrum), S32.2 (fracture of coccyx), S32.3 (fracture of ilium), S32.4 (fracture of acetabulum), S32.5 (fracture of pubis), S32.81 (fracture of ischium), S32.83 (fracture of pelvis unspecified) and S32.89 (multiple and other fractures of pelvis). Only first fractures defined by an event-free period of at least one year prior to the event were included. Therefore, data for insured persons with pelvic fractures in the first study/observation year were excluded. Furthermore, data of insured persons with first fractures marked as a follow-up diagnosis of a former fracture were excluded. For the ascertainment of first fractures we distinguished between exclusively outpatient and at some point inpatient treated fractures. First pelvic fractures, which led to hospital treatment during the whole study period, were counted as inpatient treated pelvic fractures. The decision for this classification was made with regard to the comparability to the international literature, which relies mostly on inpatient databases.

### Ascertainment of person-years

**Fig 1:**
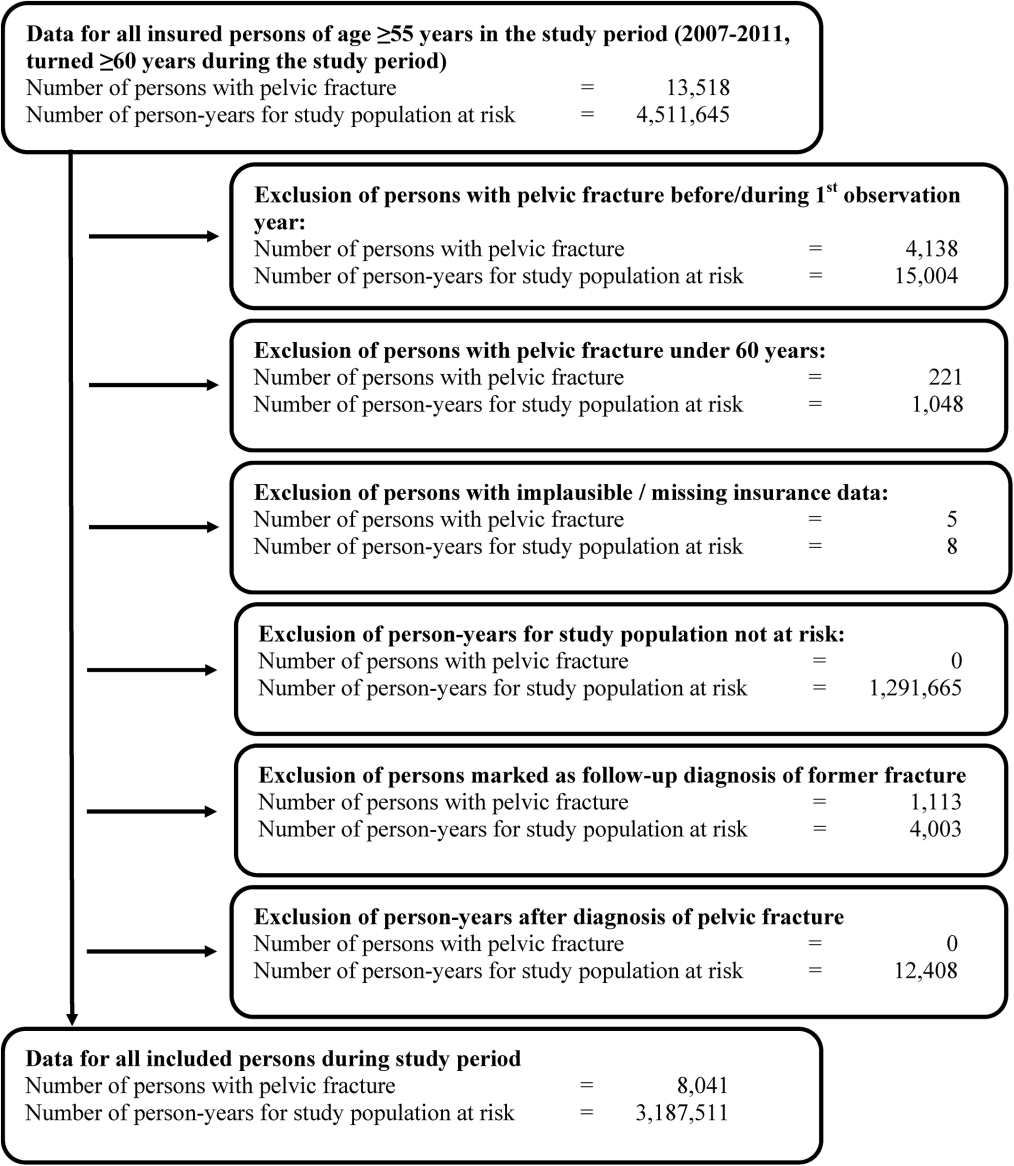
Selection process of the study population.

We calculated person-years for the individual observation periods. Person-years were summed up for all insured persons aged minimum 60 years being at risk of having a first pelvic fracture, as predefined. According to the definition, all person-years in the first year of observation were excluded. Furthermore, person-years after first pelvic fracture events were deleted. The selection process of the study population (individuals with pelvic fractures and person years) is illustrated as a flow chart in Fig 1. For the entire study population aggregated persons-times, also stratified by year, sex, age and region are provided in Table 1.

**Table 1:** Aggregated persons-time, also stratified by year, sex, age and region.

	Person-years at risk
Total [N (%)]	3,187,511 (100.0)
Calendar year [N (%)]^a^	
2008	812,797 (25.5)
2009	798,620 (25.1)
2010	790,964 (24.8)
2011	785,129 (24.6)
Sex	
Women [N (%)]	1,879,167 (59.0)
Men [N (%)]	1,308,344 (41.0)
Age in 5-year age groups [N (%)]^a^	
60–64 years	568,665 (17.8)
65–69 years	616,229 (19.3)
70–74 years	733,587 (23.0)
75–79 years	546,177 (17.1)
80–84 years	393,674(12.4)
85–89 years	228,937 (7.2)
≥90 years	100,241 (3.1)
Insured’s region^b^	
Schleswig-Holstein	937,746 (29.4)
Westfalen-Lippe	2,249,764 (70.6)

N = Number of person-years

^a^small differences from rounding of person-years might occur

^b^1 insured person in both regions, at time of first fracture in Westfalen-Lippe, counted in Westfalen-Lippe

### Further variables

We also assessed individual patient data, e.g. start and end of the period of insurance, month and year of birth and, if applicable, month and year of death, and included the following variables as possible predictors of a first pelvic fracture: age, sex, and insurance region as an approximation for the insured’s residence (Schleswig-Holstein or Westfalen-Lippe).

### Statistical analyses

Incidence rates (IR) of first pelvic fracture were calculated for the total of outpatient and inpatient events and also for inpatient treated pelvic fractures only. We estimated incidence rates (IR) per 10,000 person-years (pyrs) along with 95% confidence intervals [95% CI] by dividing the number of first fractures by the total number of person-years, overall and stratified by sex and age (5-year age groups). We also standardized incidence rates with respect to age and sex to the German population in 2009. Population data were taken from official statistics (National Office of Statistics). The association between the risk of first pelvic fracture as outcome and sex, age, calendar year and region as independent variables was examined using multiple Poisson regression, controlling for each of the aforementioned variables. We calculated estimates of relative risks (RR) along with 95% confidence intervals and corresponding p-values. To take overdispersion into account, we performed all analyses with dscale adjustment [28].

All analyses were performed using the Statistical Analysis Systems SAS (SAS for X64_8PRO, Release 9.4, SAS Institute Inc. Cary, NC, USA).

## Results

### Numbers and incidence rates of all first pelvic fractures and inpatient treated pelvic fractures

During the study period, we identified 8,041 insured persons, mostly women (82%), with first pelvic fractures. The mean age of affected insured persons was 80.3 ± 8.7 years. A total of 5,978 (74%) insured persons needed inpatient care due to pelvic fracture during the whole study period. Table 2 describes selected characteristics of persons with first pelvic fractures.

**Table 2:** Characteristics of persons with first pelvic fractures.

	Persons with first pelvic fracture
Total [N (%)]	8,041 (100.0)
Women [N (%)]	6,617 (82.3)
Men [N (%)]	1,424 (17.7)
Age (yrs) [Mean, SD]	80.3±8.7
Age in 5-year age groups [N (%)]	
60–64 years	388 (4.8)
65–69 years	610 (7.6)
70–74 years	1,136 (14.1)
75–79 years	1,372 (17.1)
80–84 years	1,733(21.6)
85–89 years	1,702 (21.2)
≥90 years	1,100 (13.7)
Outpatient treatment [N (%)]	2,063 (25.7)
Insured’s region^a^	
Schleswig-Holstein	2,210 (27.5)
Westfalen-Lippe	5,831 (72.5)

N = Number of participants; yrs = Years; SD = standard deviation

^a^1 insured person in both regions, at time of first fracture in Westfalen-Lippe, counted in Westfalen-Lippe

Table 3 shows crude incidence rates of all first fractures identified from inpatient and outpatient data and crude incidence rates for inpatient treated fractures. The crude incidence rate of all first pelvic fractures was 25.2 [95% confidence interval 24.7–25.8] per 10,000 person-years. For inpatient treated pelvic fractures the crude incidence rate corresponds to 18.8 [18.3–19.2] per 10,000 pyrs.

**Table 3:** Crude and standardized pelvic fracture incidence rates: Incidence rate per 10,000 person-years at risk and [95% confidence Interval], overall and sex-specific.

Total population aged ≥ 60years				
	Number of fractures	Person-years at risk	Pelvic fractures/10,000 pyrs [95% CI]	Standardized Incidence rate^b^
All first pelvic fractures	8,041	3,187,511	25.2 [24.7–25.8]	22.4 [22.0–22.9]
Inpatient treated pelvic fractures^a^	5,978	3,187,511	18.8 [18.3–19.2]	16.5 [16.1–16.9]
**Women**				
All first pelvic fractures	6,617	1,879,167	35.2 [34.4–36.1]	28.7 [28.0–29.4]
Inpatient treated pelvic fractures^a^	4,937	1,879,167	26.3 [25.5–27.0]	20.7 [20.1–21.3]
**Men**				
All first pelvic fractures	1,424	1,308,344	10.9 [10.3–11.4]	12.1 [11.5–12.8]
Inpatient treated pelvic fractures^a^	1,041	1,308,344	8.0 [7.5–8.4]	9.0 [8.4–9.6]

CI = Confidence Interval; pyrs = Person-years

^a^First fractures leading to hospital treatment

^b^Standard: German population 2009

### Standardized incidence rates of all first pelvic fractures and inpatient treated pelvic fractures

Table 3 also displays standardized incidence rates. The standardized incidence rate of all first fractures was 22.4 [22.0–22.9] per 10,000 pyrs. It was significantly higher in women than in men: 28.7 [28.0–29.4] vs. 12.1 [11.5–12.8] per 10,000 pyrs respectively (p < 0.001). The standardized incidence of all inpatient treated fractures was 16.5 [16.1–16.9] per 10,000 pyrs (age standardized using the whole German population in 2009, using the same basis for women and men).

### Age- and sex-specific incidence rates of all first pelvic fractures and inpatient treated pelvic fractures

**Fig 2:**
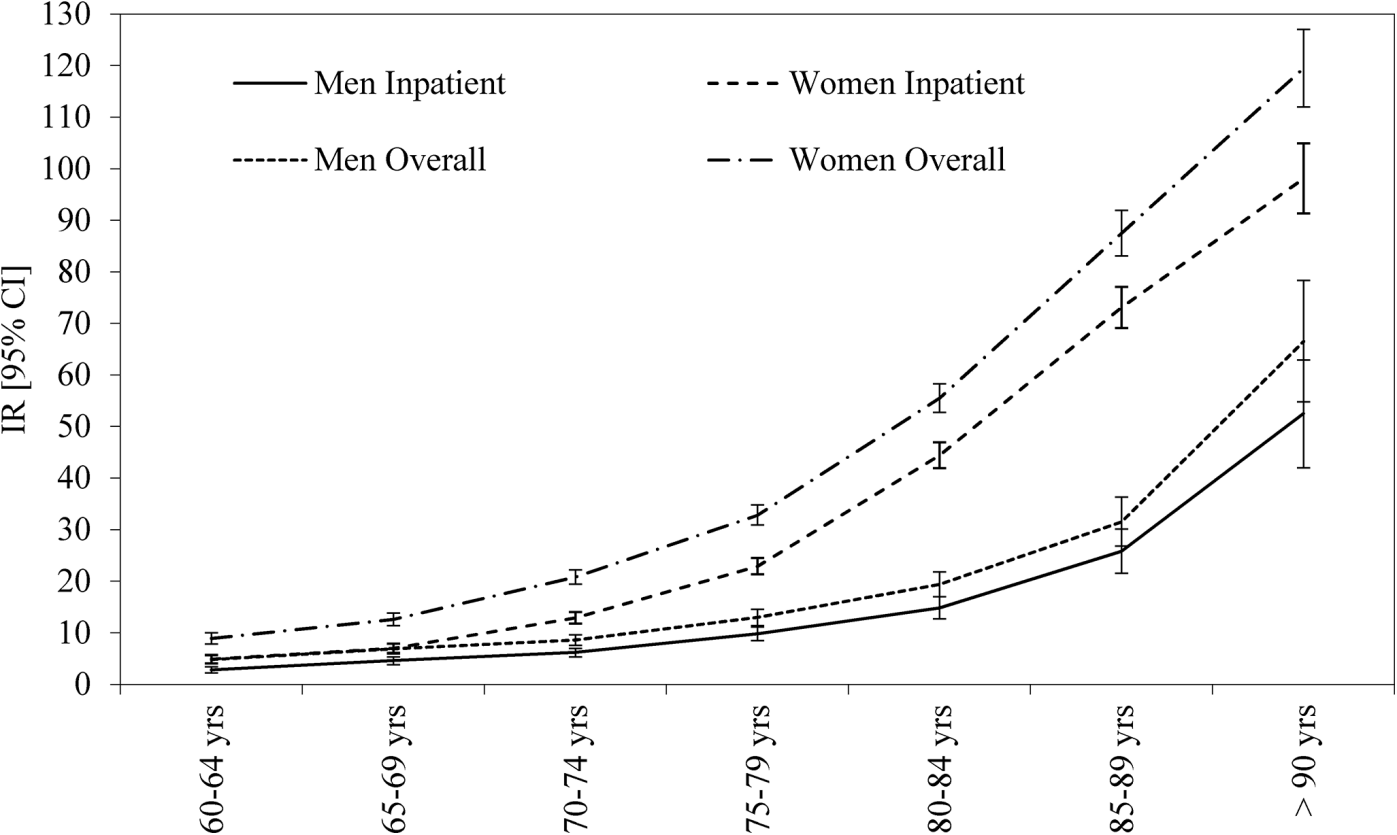
Age adjusted incidence rates of pelvic fractures per 10,000 pyrs in men and women 60 years or older (all first pelvic fractures and inpatient treated pelvic fractures).

The incidence rates of all first pelvic fractures and of inpatient treated pelvic fractures, stratified by sex and age, are illustrated in Fig 2. As expected, we found higher incidence rates in women than in men. These differences were observed in all age groups, but in particular for women in the higher age groups. Incidence rates of all first pelvic fractures and inpatient treated pelvic fractures, stratified by sex and age, are also tabulated in Table 4.

**Table 4:** Incidence rates of all first pelvic fractures and inpatient treated pelvic fractures, stratified by sex and age.

	Women			Men		
	Number of fractures	Person-years at risk	Pelvic fractures/10000 pyrs [95% CI]	Number of fractures	Person-years at risk	Pelvic fractures/10000 pyrs [95% CI]
**All first fractures (in total)**						
Age (yrs)						
60–64	251	281,770	8.9 [7.8–10.0]	137	286,895	4.8 [4.0–5.6]
65–69	409	324,697	12.6 [11.4–13.8]	201	291,532	6.9 [5.9–7.8]
70–74	861	413,325	20.8 [19.4–22.2]	275	320,262	8.6 [7.6–9.6]
75–79	1,097	334,228	32.8 [30.9–34.8]	275	211,950	13.0 [11.4–14.5]
80–84	1,490	268,349	55.5 [52.7–58.3]	243	125,325	19.4 [17.0–21.8]
85–89	1,532	175,042	87.5 [83.1–91.9]	170	53,895	31.5 [26.8–36.3]
≥ 90	977	81,756	119.5 [112.0–127.0]	123	18,485	66.5 [54.8–78.3]
**Inpatient treatment**						
Age (yrs)						
60–64	138	281,770	4.9 [4.1–5.7]	81	286,895	2.8 [2.2–3.4]
65–69	227	324,697	7.0 [6.1–7.9]	133	291,532	4.6 [3.8–5.3]
70–74	534	413,325	12.9 [11.8–14.0]	197	320,262	6.2 [5.3–7.0]
75–79	766	334,228	22.9 [21.3–24.5]	208	211,950	9.8 [8.5–11.1]
80–84	1,191	268,349	44.4 [41.9–46.9]	186	125,325	14.8 [12.7–17.0]
85–89	1,279	175,042	73.1 [69.1–77.1]	139	53,895	25.8 [21.5–30.1]
≥ 90	802	81,756	98.1 [91.3–104.9]	97	18,485	52.5 [42.0–62.9]

CI = confidence interval; pyrs = Person-years; yrs = years

### Possible predictors of a first pelvic fracture

As expected, the adjusted Poisson regression analysis showed a significant sex and age effect on first fracture risk (Table 5). Women had a considerably higher risk of first pelvic fracture than men (adjusted RR 2.38 [2.23–2.55], p < 0.001). The fracture risk was highest in persons aged 90 years or older (compared to those aged 60 to 64 years; adjusted RR 12.76 [11.13–14.63], p < 0.001). We found a slight association between calendar year and first fracture risk, indicating an increase of the incidence of pelvic fracture during the observation period, adjusted for age, sex and insured’s region. An additional model using trend variables for age and year showed significance for trend (adjusted RR = 1.04 [1.01–1.06], p = 0.003 per calendar year). In addition, there was a significant association with region showing higher incidences in Westfalen-Lippe; the effect, however, was small.

**Table 5:** Possible predictors of a first pelvic fracture: Relative Risk (RR) and [95% confidence Interval] estimated by a multiple Poisson regression model.

Variable	Relative Risk [95% CI]	*p* value
Sex		
Male	1.00 (Reference)	
Female	2.38 [2.23–2.55]	<0.001
Age (years)		
60–64	1.00 (Reference)	
65–69	1.43 [1.23–1.66]	<0.001
70–74	2.16 [1.88–2.47]	<0.001
75–79	3.36 [2.95–3.84]	<0.001
80–84	5.60 [4.92–6.38]	<0.001
85–89	8.95 [7.86–10.20]	<0.001
≥ 90	12.76 [11.13–14.63]	<0.001
Calendar year		
2008	1.00 (Reference)	
2009	1.01 [0.94–1.09]	0.771
2010	1.08 [1.01–1.17]	0.029
2011	1.10 [1.02–1.18]	0.009
Region		
Westfalen-Lippe	1.00 (Reference)	
Schleswig-Holstein	0.92 [0.87–0.98]	0.006

CI = Confidence Interval

## Discussion

### Main findings

In this retrospective population-based observational study we found a considerable risk of pelvic fractures in the German population aged 60 years and older. As expected, we observed a clear age and sex effect on the incidence of pelvic fractures in older persons. A total of 5,978 (74%) patients received hospital treatment, with a higher percentage of inpatient treatment in older people. That means that when only inpatient data is used, a relevant proportion of pelvic fractures are not taken into account and the proportion differs with age. The most important finding of our study is that the reported incidences are considerably higher than incidences described in the international literature, even if only inpatient pelvic fractures are evaluated. Our data indicate that pelvic fractures increased in Germany during calendar years 2008–2011, and that regional differences exist.

### Comparison to previous studies

In past studies over the last decades, several authors have found increasing incidences. For example, a study to determine trends of pelvic fracture-related hospitalizations among older people (population aged 65 years and older) in the Netherlands (1986–2011) found an increase of 39.7% in the age-adjusted incidence rate for women and an increase of 30.0% for men (1986: women 6.82 per 10,000 persons, men 2.83 per 10,000 persons vs. 2011: women 9.53 per 10,000 persons, men 3.68 per 10,000 persons) [6]. A trend analysis of osteoporotic pelvic fractures in Finland (1970–1997) based on data of the National Hospital Discharge Register reported the relative increase in the age-adjusted incidence of osteoporotic pelvic fractures as being 232% in women and 192% in men (1970: women 31 per 100,000 persons, men 13 per 100,000 persons vs. 1997: women 103 per 100,000 persons, men 38 per 100,000 persons) in the population aged 60 years and older [3]. In Germany, data about trends of pelvic fracture incidences are lacking, in contrast to data about trend of hip fractures [21, 29, 30].

Benzinger et al. provided recent data (from AOK Bavaria) on pelvic fracture rates in people with and without disability living both in the community and in nursing homes in Germany. However, the authors analyzed pelvic fracture cases based on hospital admission data [1]. As in our study, age and sex-specific incidence rates were considerably higher than incidence rates in other countries.

Previous international studies have documented overall incidences ranging from 1.0 to 9.2 per 10,000 person-years respectively [2, 3, 6, 7, 12, 31]. The obvious question is why are higher incidences reported for German populations? One reason might be that the higher incidence corresponds with a higher detection rate. Diagnostic procedures have improved considerably in the last two decades; there are an increasing number of computer tomography and MRI devices available and more examinations are performed [32–35]. Therefore, a combined effect of actually increasing incidence rates as well as an increasing awareness and improvements in diagnosis could be hypothesized. In 2008, 78% of all hospitals in Germany were able to carry out computer tomography examinations [34]. Lower incidence rates in other countries could partly be explained by the varying availability of computer tomography examinations [34, 36]. For the same year, a considerably larger number of CT devices were available in German hospitals (n = 1374; 16.73 per million population) in comparison to the Netherlands (n = 163; 9.91 per million population) or Spain (n = 677; 14.73 per million population) [36]. The exact reasons for the differences in incidence rates for different countries cannot fully be disentangled. It may be that different risk patterns exist in different populations. Moreover, it cannot be ruled out that methodological differences between studies account for variations in reported incidence rates.

### Implications

Pelvic fracture incidence rates turned out to be considerably higher than expected, which is a worrying sign. The predicted increase will have major effects on both individual and societal burden, on the one hand causing deterioration in mobility and increased dependency for the individual and on the other hand resulting in rising healthcare costs due to the need for hospital and follow-up care. Our results emphasize the need for preventive measures aimed at stopping the increase and stimulating a levelling off or even a decrease in pelvic fracture incidence rates, as has been reported for the occurrence of hip fractures in a number of countries. Further analyses and studies are needed to explore the factors (if any) related to different fractures or to find out which falls and fracture prevention programs are best suited. Future research should also focus on the trends of incidence rates. Monitoring of prevention programs will help to target prevention strategies.

### Strengths and limitations

Our study incorporates several important strengths. For one, we used longitudinal health insurance data of a large population-based sample in order to get valid estimates of epidemiological measures. Furthermore, health insurance data provide information of treatment in routine care. Because we had personalized individual data, we were able to describe first pelvic fractures and hence avoid overestimation due to double counting. This also meant that, when estimating predictors, bias could be prevented, which may occur since subsequent fractures are highly predicted by a previous one. After the first pelvic fracture, it is rather difficult to distinguish follow-up pelvic fractures from the follow-up therapies of the first fracture mentioned in the data. Furthermore, the risk after the first fracture might change because of successful or unsuccessful therapy or behaviour of the insured person. Therefore, other risk factors might weaken the results because of additional inhomogeneity of the data.

Several limitations have to be considered: We used only specific ICD 10 coding for pelvic fracture, since clinical expertise suggests, that codings for external causes of morbidity and mortality are not very reliable in Germany. Therefore, level of associated trauma and causes of injury were not assessed. However, we assume that in older individuals pelvic fractures are in most cases caused by low energy trauma like simple falls. In addition, with the definition of pelvic fracture according to ICD coding used in our study, we used a comparable approach as the only other German publication regarding incidence of pelvic fractures [1]. Only individual case data for the persons with pelvic fractures was provided. Person-years were aggregated for all insured persons aged 60 years or older and at risk of having a first pelvic fracture. Most persons were continuously insured with the AOK NordWest, which is in line with results of a study conducted by Hoffmann and Icks [37]. Therefore, person-years per calendar year should resemble persons in a very adequate manner. A first fracture was defined by an event-free period of one year. It has to be assumed that prior fractures may have occurred. However, the number should be small. In addition, due to the uncertainty about how long the treatment of a single pelvic fracture may last, some fractures defined as those receiving outpatient treatment may have later been treated in an inpatient setting. However, according to clinical experience misclassification may be low. Moreover, it has to be taken into account that AOK members are not representative for the whole German population. They have been found to be older and more likely to be socially deprived compared to members of other health insurances [38]. In this study by Hoffmann and Icks structural differences between statutory health insurance companies and their impact on health service research were assessed. With regard to the prevalence of chronic diseases, the data showed considerable differences between the various German health insurance companies. However, in our study we standardized the incidence rates for the German population, adjusting partially for age and sex deviations from the general German population. Nevertheless, the generalization of our findings for other populations has to be proven.

### Conclusions

We estimated incidence rates of pelvic fractures in the German population aged 60 years or older based on outpatient and inpatient data from a health insurance company. Incidence rates are considerably higher than incidences described in the international literature. If data is available, inpatient and outpatient events should be analyzed; otherwise a relevant proportion of pelvic fractures are not taken into account. We further evaluated the association between the risk of first pelvic fracture as outcome and sex, age, calendar year and region as independent variables and found that the considerably high incidences observed were significantly influenced by sex and age. There may be an increase in the risk of pelvic fractures due to calendar years and regional differences. The latter has to be confirmed in future studies.

Our study results are highly relevant for policy makers who have to make decisions on health service planning and prevention, such as fall prevention programs.
